# Predicting Human Nucleosome Occupancy from Primary Sequence

**DOI:** 10.1371/journal.pcbi.1000134

**Published:** 2008-08-22

**Authors:** Shobhit Gupta, Jonathan Dennis, Robert E. Thurman, Robert Kingston, John A. Stamatoyannopoulos, William Stafford Noble

**Affiliations:** 1 Department of Genome Sciences, University of Washington, Seattle, Washington, United States of America; 2 Department of Molecular Biology, Massachusetts General Hospital, Boston, Massachusetts, United States of America; 3 Division of Medical Genetics, University of Washington, Seattle, Washington, United States of America; 4 Department of Computer Science and Engineering, University of Washington, Seattle, Washington, United States of America; University of California San Diego, United States of America

## Abstract

Nucleosomes are the fundamental repeating unit of chromatin and comprise the structural building blocks of the living eukaryotic genome. Micrococcal nuclease (MNase) has long been used to delineate nucleosomal organization. Microarray-based nucleosome mapping experiments in yeast chromatin have revealed regularly-spaced translational phasing of nucleosomes. These data have been used to train computational models of sequence-directed nuclesosome positioning, which have identified ubiquitous strong intrinsic nucleosome positioning signals. Here, we successfully apply this approach to nucleosome positioning experiments from human chromatin. The predictions made by the human-trained and yeast-trained models are strongly correlated, suggesting a shared mechanism for sequence-based determination of nucleosome occupancy. In addition, we observed striking complementarity between classifiers trained on experimental data from weakly versus heavily digested MNase samples. In the former case, the resulting model accurately identifies nucleosome-forming sequences; in the latter, the classifier excels at identifying nucleosome-free regions. Using this model we are able to identify several characteristics of nucleosome-forming and nucleosome-disfavoring sequences. First, by combining results from each classifier applied de novo across the human ENCODE regions, the classifier reveals distinct sequence composition and periodicity features of nucleosome-forming and nucleosome-disfavoring sequences. Short runs of dinucleotide repeat appear as a hallmark of nucleosome-disfavoring sequences, while nucleosome-forming sequences contain short periodic runs of GC base pairs. Second, we show that nucleosome phasing is most frequently predicted flanking nucleosome-free regions. The results suggest that the major mechanism of nucleosome positioning in vivo is boundary-event-driven and affirm the classical statistical positioning theory of nucleosome organization.

## Author Summary

Inside the nucleus, DNA is wrapped into a complex molecular structure called chromatin, whose fundamental unit is ∼150 bp of DNA organized around the eight-histone protein complex known as the nucleosome. Understanding the local organization of nucleosomes is critical for understanding how chromatin impacts gene regulation. Here, we describe a computational model that predicts nucleosome placement from DNA sequence. We train the model using data derived from human cell lines, and we apply the model systematically to 1% of the human genome. We show that previously described models trained from yeast data correlate strongly with the human-trained model, suggesting a common mechanism for sequence-based determination of nucleosome occupancy. In addition, we observe a striking complementarity between models trained using data from weakly and strongly digested samples: one type of model recognizes nucleosome-free regions, whereas the other identifies well-positioned nucleosomes. Finally, our analysis of predicted nucleosome positions in the human genome allows us to identify common features of nucleosome-forming and inhibitory sequences. Overall, our results are consistent with the classical statistical positioning theory of nucleosome organization.

## Introduction

Nucleosomes are the fundamental repeating unit of chromatin, and the positioning of nucleosomes along the genome has been a topic of long-standing interest. The prevailing “statistical positioning” theory of nucleosome organization was first proposed by Kornberg more than 25 years ago [1]. This theory, for which considerable experimental evidence exists [2], posits that nucleosomes are stochastically positioned along the genome and are distributed between boundary events that comprise nucleosome-free regions, such as those known to be found at the promoters of active or poised genes.

According to statistical positioning theory, the repetitive nucleosomal structure is dynamically punctuated by short regions where regulatory factors bind in place of canonical nucleosomes. Whether a particular genomic position is occupied by a nucleosome may therefore vary from cell to cell within a population of cells and between different cell types. However, it is expected that the vast majority of the genome at any given time is covered by nucleosomes. The observation that specific DNA sequences favored the formation of nucleosomes [3]–[10] raises the possibility that sequence plays a significant role in organizing nucleosomal arrays in vivo.

The determination of nucleosome placement along the genome presumably depends upon a variety of factors, including properties of the sequence itself, physical constraints, and epigenetic factors such as ATP-dependent chromatin remodeling or alterations in the biochemical composition of the histone octamer. MNase cleaves chromatinized DNA preferentially in inter-nucleosomal linker regions; with robust digestion chromatin can be reduced to mononucleosomes and their associated ∼147 bp DNA fragments, which can in turn be mapped to the genome to reveal nucleosome positions using either tiling DNA microarrays or sequencing assays.

Recently, Segal et al. [11] proposed a computational model for sequence-based prediction of nucleosome positioning in *S. cerevisiae*. The model is generative and, motivated by previous work [12], uses dinucleotide frequencies collected from a training set of aligned nucleosome-bound sequences. Segal et al. used dynamic programming to identify the highest-scoring series of nonoverlapping nucleosome positions along a given chromosome, and demonstrated that, for a test set of nucleosomal sequences not used in the training procedure, the model places 54% of the nucleosomes within 35 bp of their true locations. This is 15% higher than would be expected by random placement of the same number of nucleosomes along the genome.

Subsequently, Peckham et al. [13] proposed a complementary computational model that is discriminative, rather than generative, and that focuses only on sequences that show the strongest signals of nucleosome occupancy or vacancy in a microarray-based assay of *S. cerevisiae* [14]. Briefly, the assay consists of cleaving nucleosomal and bare genomic DNA with MNase, and then measuring the abundance of uncleaved products using a tiling microarray with 50-mer probes. The model of Peckham et al. is trained to discriminate between microarray probe sequences that showed the highest and lowest fluorescence log-ratios. The resulting model correctly predicts 50% of well-positioned nucleosomes within 40 bp of their correct positions. Like the model of Segal et al., this represents an improvement of 15% relative to random placement of nucleosomes along the genome. Recently, two variants of the MNase microarray assay were applied to human DNA [15],[16]. Here, we demonstrate that the discriminative model described by Peckham et al. can be successfully trained from these two human datasets. We chose to use the discriminative approach because the current understanding of chromatin biology suggests that there are, indeed, genomic sequences which ensure nucleosomal occupancy [11],[14], as well as sequences which nucleosomes seem to avoid. The latter include nucleosome-free regions enriched in gene regulatory regions, CpG islands, and transcription termination sites [14], [17]–[19], nucleosome-free regions flanked by H2A.Z [20],[21], nucleosome-free regions ∼200 bp upstream of the start codon of Pol II transcribed genes in yeast [14],[21], polycomb response elements [22],[23], and P53 binding sites [24]. Because several lines of inquiry indicate that primary genomic sequences can positively or negatively influence whether a particular locus is nucleosomally occupied, we felt justified in using the SVM to discriminate between probes in our datasets that were more or less MNase sensitive and thus more or less likely to be occupied by nucleosomes.

We find that, for both of the human MNase array datasets that we investigated, the SVM model achieves a comparable level of cross-validated accuracy as the yeast-trained model. Furthermore, we observe that the qualitative behavior of the learned model depends strongly the amount of MNase digestion applied to the given sample. At lower levels of digestion, the model excels at recognizing regions that are protected from MNase cleavage; conversely, with stronger digestion, the model accurately identifies regions of accessibility to MNase cleavage. Essentially, the former model predicts the presence of a strongly positioned nucleosome, whereas the latter predicts the absence of a nucleosome. Next, we apply both types of trained model to the ENCODE regions of the human genome, and we observe close agreement with the yeast-trained models of Peckham et al. and Segal et al., suggesting a shared mechanism for sequence-based determination of nucleosome occupancy. Additionally, our model indicates that nucleosomal occupancy is primarily determined by short genomic sequences, with C- and G-containing dinucleotide pairs strongly represented in the nucleosome-forming sequences. We also show that the models predict a dip upstream and a peak downstream of annotated transcription start sites in the ENCODE regions, suggestive of nucleosome placement at the TSS with nucleosome absence immediately upstream. The model was also used to understand the characteristics of sequences around nucleosome-forming and nucleosome inhibitory probes. We find that nucleosome-forming probes do not necessarily prescribe a translationally positioned nucleosome, while sequences surrounding nucleosome inhibitory sequences have SVM discriminant scores that indicate translationally positioned nucleosomes. Finally, the model can be used to make predictions of nucleosomal occupancy on DNA fragments used in in vitro experiments. We can use these predictions to refine our understanding of the primary sequence contribution to ATP-dependent remodeling experiments.

Overall, our results suggest that, to the extent that nucleosome positioning signals exist, they exist in tandem with nucleosome-free regions. This is fundamentally consistent with the statistical positioning theory, in which nucleosome positioning and nucleosome-free region boundary events are mechanistically linked.

## Results

### Cross-Validated Testing of Nucleosome Occupancy Predictors

We began our analyses by training a model using the microarray data from Dennis et al. [16]. This dataset contains three microarrays, each containing ∼120,000 probes. The probes cover 25 kb regions upstream of 42 genes, using 50-mer probes tiled every 20 bases. For all of our analyses, we omitted probes that overlap repetitive elements, as identified by RepeatMasker [25]. Each 50-mer is included on the array three times, as is its reverse complement. Thus, three replicate arrays yield 18 measurements per 50-mer. To train the model, we followed the protocol described by Peckham et al., first identifying the top 1,000 and bottom 1,000 probes by log ratio (see Methods). Each probe sequence is then represented as a 2,772-element vector, in which each entry is a normalized count of the occurrences of a particular *k*-mer or its reverse complement, for *k* = 1 up to 6. These vectors were used to train a support vector machine [26], which is a powerful discriminative classification algorithm that is widely used in diverse bioinformatics applications [27].

We evaluated the quality of the resulting classifier using a cross-validation procedure. In this procedure, the dataset is divided at random into ten subsets. An SVM is trained on 90% of the data (i.e., using 1,800 probes) and tested on the held-out probes. This train–test procedure is repeated ten times using a different hold-out set each time.

**Figure 1:**
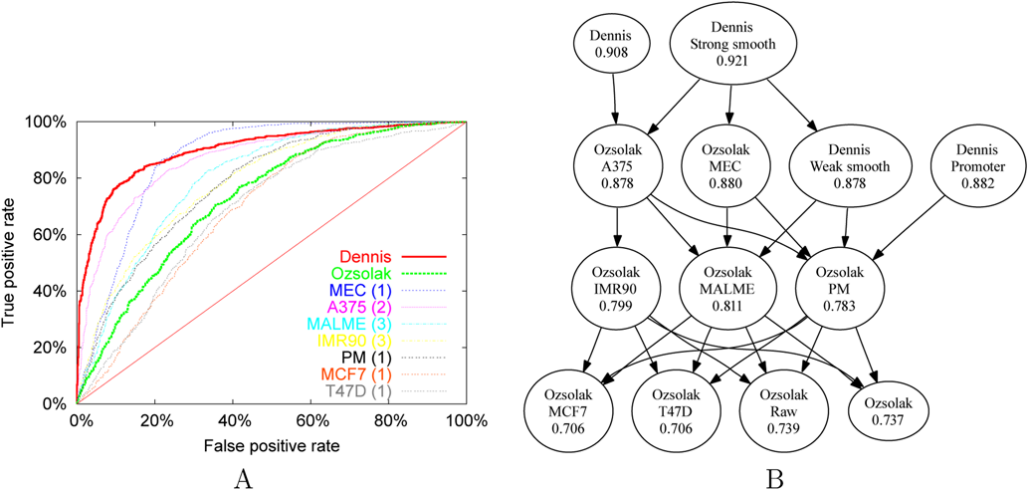
Predictive ability of the SVM classifiers. (A) The figure plots the rate of true positive identifications as a function of the rate of false positive identifications, computed for varying thresholds on the SVM discriminant. The series correspond to SVMs trained and tested using probes from the Dennis et al. dataset (“Dennis”), the entire Ozsolak et al. dataset (“Ozsolak”), and from seven individual cell lines within the Ozsolak dataset. In the legend, the numbers in parentheses indicate the number of arrays performed for each cell line. (B) The figure shows the results of pairwise comparisons among the various SVMs. In the graph, each node corresponds to an SVM, and a directed edge from *A* to *B* indicates that SVM *A* performs better than SVM *B* according to a Wilcoxon signed-rank test, *p*<0.01. Redundant edges have been removed from the graph; i.e., if *A* is better than *B*, and *B* is better than *C*, we remove the edge from *A* to *C*. Each node is labeled with the name of the SVM and the corresponding median ROC score.

Figure 1 shows that the SVM trained on the microarray data from Dennis et al. is strongly predictive. The figure shows a receiver operating characteristic (ROC) curve [28], which plots the rate of true positives as a function of the rate of false positives for varying classification thresholds. To produce the ROC curve, each probe in the test set is ranked according to the discriminant score produced by the SVM. In this ranked list, a random classifier would not successfully separate positive from negative probes and would therefore produce a diagonal line at approximately *y* = *x*. On the other hand, a perfect classifier would rank all positive probes above all negative probes, producing a line that travels from the origin vertically to (0,1) and then horizontally to (1,1). The quality of a classifier can be evaluated by computing the area under the ROC curve (the “ROC score”), with 0.5 corresponding to chance and 1.0 corresponding to perfect separation. For the SVM trained on probes from the Dennis et al. dataset, the median ROC score across the ten cross-validations is 0.908, which is significantly better than chance. In a similar ten-fold cross-validation experiment, Peckham et al. report an ROC score of 0.951 from a similar SVM trained on the yeast dataset of Yuan et al. [14].

Next, we repeated the SVM cross-validation testing procedure using data generated by Ozsolak et al. [15]. As before, we selected the top 1,000 and bottom 1.000 probes for training and testing the SVM. Ozsolak et al. performed their analyses on seven different cell lines. Therefore, we trained seven different SVMs using data from each cell line. We also trained an eighth SVM by selecting probes that showed a consistently high or low level of fluorescence intensity across cell lines, using a rank-based selection procedure (see Methods). All eight ROC curves are included in Figure 1A. We compute the statistical significance of differences in ROC scores using the Wilcoxon signed-rank test. Figure 1B shows the results of pairwise comparisons among all of the SVMs, using a threshold of *p*<0.01.

Each of the eight SVMs performs significantly better than chance. However, the predictive power of the SVMs trained on different cell lines varies dramatically, from a median ROC score of 0.706 for T47D and MCF7 up to 0.880 for MEC. This variance does not correlate with the amount of replicate data available. The Ozsolak et al. dataset contains triplicate arrays for two of the cell lines (MALME and IMR90) and duplicate arrays for one cell line (A375). However, the best-performing SVM—MEC and A375, with median ROC scores of 0.880 and 0.878, respectively—are based on one and two microarrays, respectively. As shown in Figure 1B, these two SVMs perform significantly better than all of the other six SVMs.

### MNase Cleavage Differences in SVM Training Sets Reveal Complementary Aspects of Chromatin Accessibility

Even between these two best-performing SVMs, the qualitative performance of the classifier differs significantly. This difference can be seen in the shapes of the respective ROC curves in Figure 1A. For MEC, the ranked list of test set probes contains a significant proportion of false positives near the top of the list, and very few false negatives near the bottom of the ranked list. Conversely, the SVM trained on data from the A375 cell line maintains a low false positive rate near the top of the list, but suffers a higher false negative rate lower down the list. Thus, the MEC SVM is good at recognizing regions of high accessibility to MNase, whereas the A375 is good at recognizing regions of strong protection from MNase cleavage. This characterization agrees well with the observation that the A375 cell line was digested with a different, less potent batch of MNase than was used for all the other cell lines [15].

Surprisingly, the SVM trained on all seven cell lines performs worse than five of seven SVMs trained on data from individual cell lines. A priori, an attractive model is that local patterns of nucleosome protection and exposure can be divided into tissue-specific and constitutive patterns. Presumably, the nucleosome positioning signal in regions that show consistent patterns across tissues would be encoded in the genome, which is static, whereas extra-genomic signaling would control tissue-specific positioning. Contrary to this model, the large variability in predictability among different cell lines and in particular the relatively poor performance of the SVM trained on probes that are consistently high or low across cell lines suggest that constitutive patterns of nucleosome protection and exposure are not preferentially encoded in the genome.

### Systematic Differences between the Datasets Do Not Affect the Performance of the SVM

Overall, the SVM trained on the data of Dennis et al. performs much better than the SVM trained on the complete Ozsolak dataset, but performs comparably to the best-performing SVM trained on a single cell line. To determine whether the strong performance of the Dennis SVM can be explained by a systematic difference between the two datasets, we performed several additional experiments.

One important difference between the two microarray datasets that we investigated lies in the design of their probes. The Dennis et al. data spans −20 kb to +5 kb around the transcription start sites of genes selected based on their transcriptional response to ATP-dependent chromatin remodelers, whereas the Ozsolak et al. data covers only −1,250 to +250 bases around the transcription start sites of human cancer-related and randomly selected genes. If promoter regions contain systematic differences in nucleosome positioning relative to larger upstream regions, then these differences will be apparent in the two datasets.

Therefore, to reduce the differences between the Dennis SVM and the Ozsolak SVM, we trained an additional SVM. This time, we used probes from the Dennis et al. dataset, but we only considered probes that lie in the promoter-proximal region, as defined by Ozsolak et al. (i.e., −1,250 to +250 bases from the transcription start site). To ensure that we retain a similar degree of nucleosome protection or exposure as the initial Dennis SVM training set, we selected only the 150 probes with highest and lowest intensity, rather than 1,000 probes for each class. The resulting promoter-specific SVM performs slightly worse than the original Dennis SVM, achieving a median ROC score of 0.882. This is not significantly different from the ROC score of the original Dennis SVM, and it is comparable to the performance of the MEC, A375 and MALME SVMs.

A second difference between the Dennis and Ozsolak datasets is related to data processing. A significant concern with any microarray study is the possibility of bias resulting from probe hybridization artifacts. To combat this potential bias, Ozsolak et al. employ a wavelet denoising procedure which attempts to identify probes that show significantly higher or lower signal than either of their flanking probes. Dennis et al. did not perform any such denoising. It is therefore possible that our SVM performs well on the Dennis dataset precisely because it is able to learn to recognize these hybridization artifacts.

To further investigate whether the SVM is learning to recognize hybridization artifacts, we trained three additional SVMs. The first SVM is trained on the raw Ozsolak data, prior to wavelet denoising. The second and third SVMs are trained on wavelet smoothed versions of the Dennis dataset (“strong” smoothing and “weak” smoothing; see Methods). In all three cases, wavelet smoothing has no significant effect on the cross-validated ROC score. For the Ozsolak SVM, removing the wavelet smoothing changes the median ROC from 0.737 to 0.739. For the Dennis SVM, weak smoothing causes the median ROC score to decrease slightly, and strong smoothing causes the median ROC score to increase slightly. None of these differences is statistically significant. Thus, it does not appear that probe hybridization artifacts explain the difference in performance between the Ozsolak and the Dennis SVMs.

### The SVM Agrees with Predictions Made by Models Trained on Yeast Data

We next sought to determine whether yeast and human share common nucleosome positioning primary sequence features. Toward this end, we used the ENCODE regions, which span 1% of the human genome and systematically cover a range of gene densities and densities of conserved noncoding sequence. We tiled the ENCODE regions with nonoverlapping 50-mer probes and computed corresponding discriminant scores from the SVM trained using the A375 cell line of the Ozsolak dataset. We used the A375 SVM because, as shown above, this SVM excels at recognizing regions of strong nucleosome protection. We also applied two yeast-based models to these same probes: an SVM trained on the yeast microarray data from Yuan et al. [14], following the protocol of Peckham et al. [13], and the dinucleotide position-specific scoring matrix (PSSM) described in [11]. For the latter, we use 138 bp centered around each 50-mer probe, and we do not include the dynamic programming portion of the Segal et al. method.

**Figure 2:**
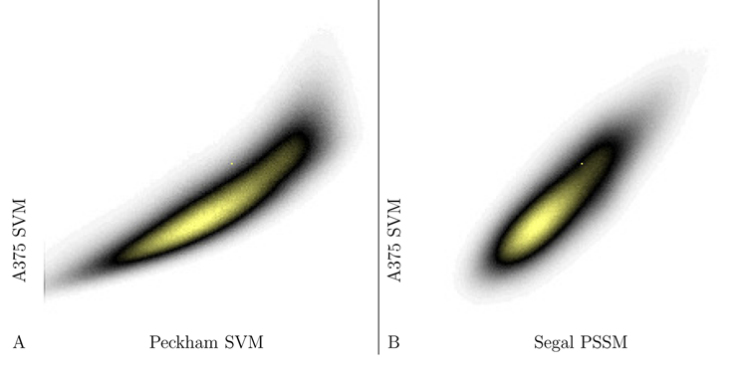
Predicted nucleosome occupancy in human and yeast. Each panel plots the predicted nucleosome occupancy from the SVM trained on human data as a function of the predicted occupancy from a model trained using yeast data.

The predictions made by the A375 SVM trained on human data correlate strongly with the predictions made by both of the models trained on yeast data. Figure 2 shows density heatmaps of scatter plots of predicted nucleosome positioning for human versus yeast models. The observed correlations for A375 are 0.862 (Peckham model) and 0.849 (Segal model). The corresponding correlations for the Dennis SVM (not shown), which also recognizes nucleosome protected regions, are 0.879 and 0.847. These correlations strongly suggest that the human-trained and yeast-trained models are learning to recognize a common sequence pattern.

### Top- and Bottom-Scoring Probes Show Distinct Composition and Periodicity Features

**Figure 3:**
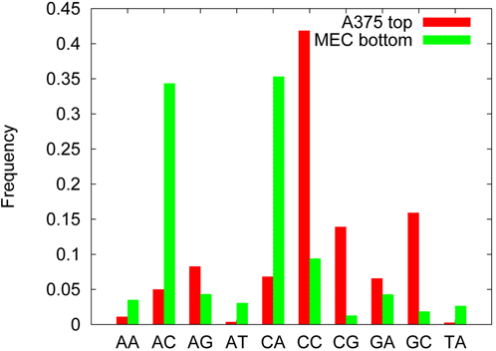
Dinucleotide frequencies among top- and bottom-ranked probes. The figure plots the frequency of each dinucleotide among the top-ranked probes from the A375 SVM and the bottom-ranked probes from the MEC SVM.

DNA primary sequence plays a large role in the conformation of the double helix. More than 20 years ago, Drew and Travers [29] showed that certain dinucleotide frequencies are amenable to deformation around the nucleosome core. Many similar papers have since addressed the location and frequency of dinucleotide pairs in nucleosome formation. In order to understand the features of sequences that make them more or less suitable for organization around the histone octamer, we collected the 1,000 highest scoring probes identified by the A375 SVM within the ENCODE regions, and a complementary set of 1,000 lowest scoring probes identified by the MEC SVM (see Methods for details). These probes represent nucleosome-forming sequences and nucleosome inhibitory sequences, respectively. Compared to the training set probes, the ENCODE 50-mers represent a much smaller proportion of the genome—0.033% of the 30 Mbp ENCODE regions, compared to 0.9% and 1.8% of the probes on the Dennis and Ozsolak arrays, respectively. Also, unlike the microarray probes, the ENCODE 50-mers are not restricted to promoter-proximal regions of the genome. We analyzed dinucleotide usage within each of the ENCODE probe sets (Figure 3). The sequences identified by the A375 SVM as nucleosome-forming sequences are rich in CC/GG dinucleotides (41.8%). Conversely, AA/TT, AT, and TA sequences are severely underrepresented in these probes, collectively accounting for only 1.7% of the dinucleotides. One concern when using datasets generated from MNase cleavage involves the slight sequence bias of MNase for AT sequences (Wingert and VonHippel, 1968). However, this sequence preference is unlikely to account for the vast differences seen in the AT underrepresentation in the nucleosome-forming probes. Among the bottom ranked, nucleosome inhibitory sequences, we find a clear overrepresentation of AC/GT and CA/TG dinucleotides (69.7%).

**Figure 4:**
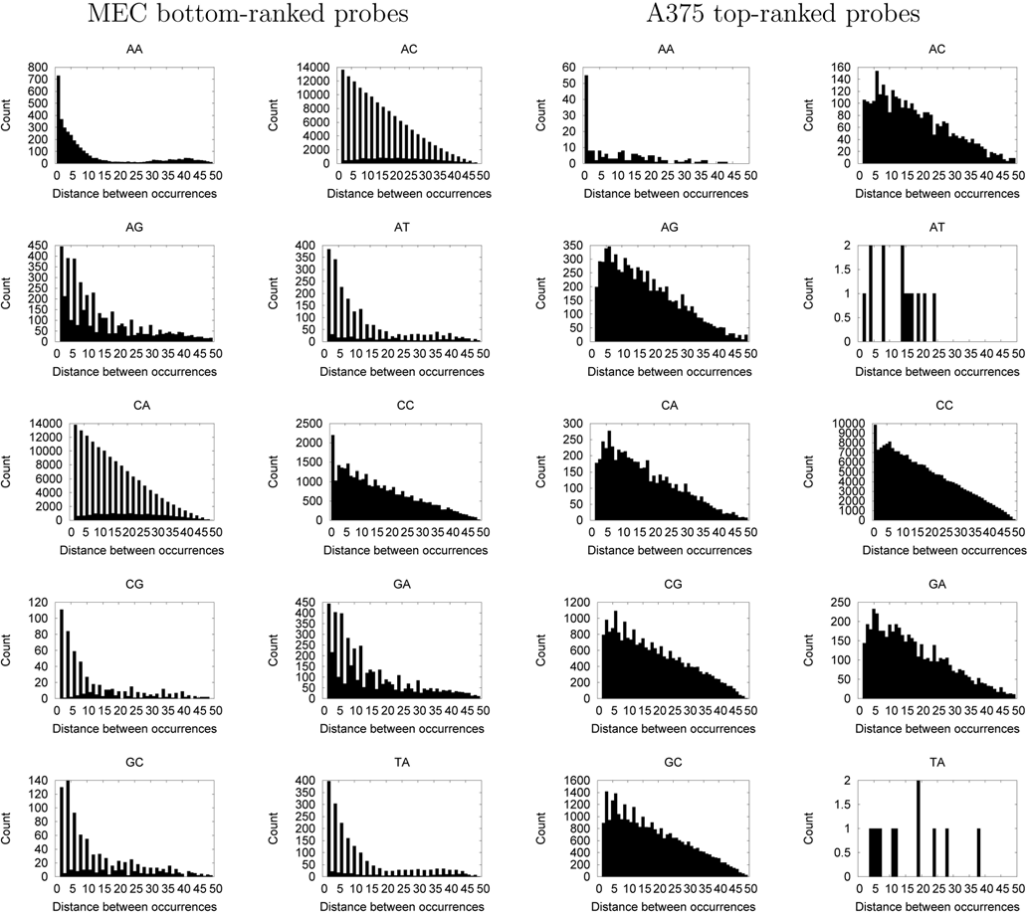
Periodicity of dinucleotide frequencies among top- and bottom-ranked probes. Each figure plots the count (*y*-axis) of identical pairs of dinucleotides at a given spacing (*x*-axis) within a given set of probes. The two left columns of panels contain dinucleotide counts from the bottom-ranked probes identified by the MEC SVM; the two right columns of panels contain counts from the top-ranked probes identifed by the A375 SVM.

The observations regarding dinucleotide abundance prompted us to analyze the periodic nature of their occurrence. We computed the distance between a given dinucleotide and each of its identical neighbors and then plotted counts of all pairwise distances in a single histogram for each dinucleotide (Figure 4). Analysis of the top ranked nucleosome-forming sequences reveals a clear 3 bp periodicity of CG and GC dinucleotides with a distance of 3 bp. Three base pairs represents about one third of a helical turn of the DNA helix, and periodic occurrences of GG/CC and GC dinucleotides have been implicated as a nucleosome positioning signal in human DNA [30],[31].

The GC 3 bp periodicity is particularly interesting because many lines of evidence implicate trinucleotide repeats of CTG and CAG as potent nucleosomal occupancy positioning signals. For example, CTG repeats do not appear to have any rotational phasing preference with respect to the core nucleosome [32], and relatively straight DNAs characterized by CTG repeats have a high affinity with the histone octamer in reconstitution experiments [33],[34]. Furthermore, sequences of DNA sequences containing 75 or 130 CTG repeats were shown to form nucleosomes 6 and 9 times more strongly, respectively, than the 5s rDNA, a naturally occurring nucleosome positioning sequence [35]. Surprisingly, DNAse I experiments have indicated that CTG repeats are among the most flexible trinucleotides, indicating that this relatively straight DNA has the potential to bend [36]. CAG repeats, on the other hand, were found to be enriched in nucleosome positioning sequences from the mouse genome [37] and are found frequently within centromeric satellite repeats. Perhaps most significantly, a crystal structure of the nucleosome was solved using a palindromic alpha satellite DNA sequence [38] which contains many degenerate forms of CNG runs. These CNG-type runs occur on one edge of the histone octamer, where they hug the form of the nucleosome along positions in the structure where a sharp DNA-deforming bend is not required. The location of these runs is consistent with all of the above observations. It therefore seems reasonable that the lack of rotational phasing preference, strong binding characteristics and inherent flexibility would make sequences containing this type of repeat nucleosome forming. Additionally, because this type of trinucleotide repeat does not appear to have any rotational phasing with respect to the nucleosome core particle, it is not surprising that our periodicity analysis does not reveal any 10 bp periodicity within these sequences.

Low complexity DNA sequence appears to be the hallmark of the nucleosome inhibitory probes. Eight of the ten dinucleotide periodicities appear as simple dinucleotide repeats. This is interesting in light of the fact that these probes were selected from a repeat masked library, indicating that short stretches of simply repeating sequence may have been evolutionarily constrained to ensure a fluid DNA chromatin structure at particular loci. Additionally, for several of these low complexity repeats—AG, AT, CG, GA, GC, and TA—the simple dinucleotide repeat persists for runs of less than 15 bases, indicating that a relatively short stretch of dinucleotide repeat appears sufficient to destabilize nucleosomal organization of the DNA sequence. This behavior was predicted by Drew and Travers [29] when they postulated that runs of homopolymer AT or GC would be excluded from the central region of nculeosomes due to their relative inflexibility.

### Low Scoring Probes Are Flanked by Nucleosome-Sized Regions of High-Scoring Probes

We next aimed to characterize the nucleosome formation potential of the sequences flanking the low scoring probes. Nucleosome-free regions in chromatin arise secondary to the cooperative binding of sequence-specific DNA binding factors [39]. The degree to which the primary DNA sequence contributes to this process beyond supplying the protein binding motifs is unknown. If certain DNA sequences are less stable in the context of a nucleosome (“nucleosome-excluding sequences”), then these regions would presumably provide more fertile ground for the nucleation of transcription factors.

**Figure 5:**
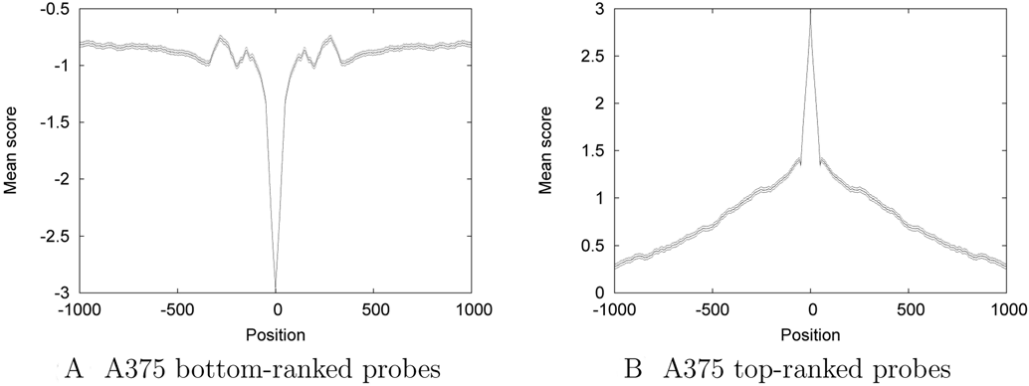
SVM discriminant scores upstream and downstream of high and low scoring probes. Each panel plots the average SVM discriminant score across the (A) top- and (B) bottom-scoring probes from the A375 and MEC SVMs, respectively, in a 2 KB region centered around the probe.

Every nucleosome-depleted region should be, by definition, flanked by nucleosome-occupied regions. Indeed, efficient positioning of nucleosomes may potentiate the coalescence of regulatory factors in the intervening region by providing higher-order stability following loss of the central nucleosome. We therefore next investigated nucleosome-disfavoring sequences in the context of their local chromatin environment. In particular, we wanted to investigate if these sequences were flanked by regions with higher than average nucleosome stabilizing sequences. Consequently, for this analysis, we used the A375 SVM, which identifies such nucleosome-forming sequences with high accuracy. We aligned the 1,000 lowest scoring probes from the A375 SVM, averaged the scores and symmetrized them to remove strand-specific artifacts. The results are striking (Figure 5, left panel): the nucleosome inhibitory sequence is flanked by probes whose discriminant scores oscillate, suggestive of two flanking well-positioned nucleosomes. These results echo what has been seen in the nucleosome-free region of yeast promoters: the nucleosome-free region is surrounded on either side by regularly spaced nucleosomes [14]. A similar oscillatory pattern is observed when we produce these plots using the Dennis SVM, but not when we use the MEC SVM (Figure S1). The absence of this pattern in the latter case presumably arises because the MEC SVM only recognizes nucleosome inhibitory sequences, rather than nucleosome-forming sequences.

### High Scoring Probes Are Not Part of a Pattern That Reflects Regularly Spaced Nucleosomes

Because nucleosome inhibitory sequences are flanked by sequences that have high nucleosome formation potential, we next sought to understand the characteristics of sequences adjacent to high scoring probes. In particular, we wanted to investigate if these sequences were set in the center of a particular pattern of nucleosome formation potential. We aligned the top-scoring 1,000 probes collected previously, and plotted average symmetrized scores, as before. In Figure 5, right panel, we see a linear dropoff on either side of the probe and no periodic information. These results indicate that a high scoring probe does not confer a pattern of nucleosomal occupancy in the surrounding sequence. The linear dropoff to baseline, as well as the magnitude of the SVM discriminant score in the flanking region, indicates that these probes are found in regions of relatively higher nucleosomal formation potential. These probes may be critical in facilitating nucleosomal occupancy at these particular locations, with the histone octamer likely adopting many positions over a window greater than 150 bp.

### Regions Upstream of Transcription Start Sites Show Markedly Lower Predicted Occupancy

**Figure 6:**
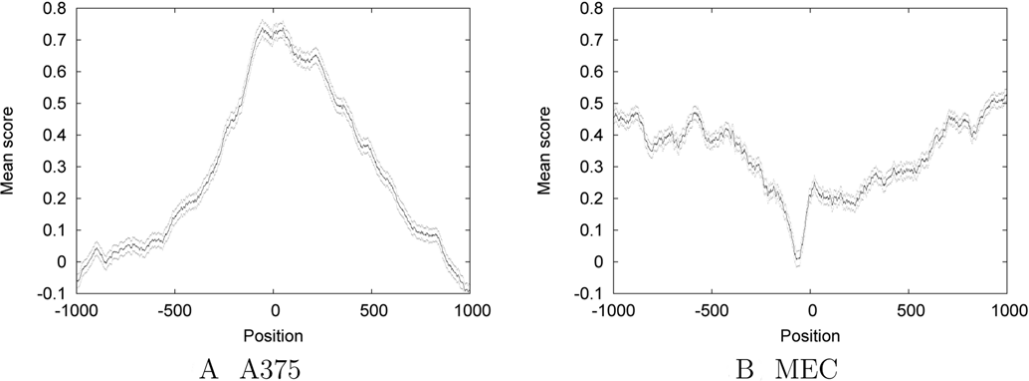
Predicted nucleosome occupancy near transcription start sites Each panel plots the average SVM-predicted nucleosome occupancy in a 1 kb region centered around Gencode-annotated transcription start sites. Flanking dots at each base position indicate the standard error of the mean.

An orthogonal means of validation is to examine the pattern of predicted nucleosome occupancy near genomic landmarks that are known to impact nucleosome positioning. We therefore examined the pattern of predicted nucleosome occupancy near transcription start sites. We expect nucleosome occupancy to decrease on average in promoter regions, because bound nucleosomes can impede promoter activity. We collected 2 kb regions centered around transcription start sites identified in the Gencode annotation [40]. Figure 6 shows, for the A375 and MEC SVMs, the average predicted nucleosome occupancy in these 1 kb regions. The A375 SVM shows a pronounced peak over the start of the gene; conversely, the MEC SVM shows a very strong dip just upstream of the TSS. A pattern similar to that produced by the MEC SVM is observed for the other six cell lines (data not shown), all of which were digested with the same batch of MNase as the MEC cell line. These results are consistent with a model in which the start of the gene is occupied by a well positioned nucleosome, which has the potential to become nucleosome-free upon gene activation. The A375 SVM recognizes the former effect, and the MEC SVM recognizes the latter.

Oszolak et al. further partitioned the genes on their array into those that are expressed and those that are not expressed in A375 cells. When the average log_2_ nucleosomal/bare genomic ratios of each of these promoter subsets are aligned at transcription start sites an interesting pattern emerges. The unexpressed genes show a nucleosomal occupancy profile very similar to that of the A375 SVM model: higher nucleosomal occupancy at the transcription start site with relatively lower occupancy in the promoter region. It is only in the expressed genes that a chromatin structure reflective of a nucleosome-free region flanked by positioned nucleosomes is seen. This is in keeping with a nucleosome remodeling event regulating gene expression.

We next sought to understand if the high scoring probes were enriched proximal to transcription start sites. In order to accomplish this we used our 1,000 top and bottom scoring probes from the ENCODE regions and plotted their proximity to transcription start sites from the Gencode annotation. For the A375 trained SVM, 589 out of the top 1,000 high scoring probes lie within 1 kb upstream or downstream of a transcription start site.

The above results are in keeping with whole genome studies that find lower nucleosomal occupancy in promoter regions than coding regions [17],[19],[41]. Our results indicate a higher potential for nucleosomal occupancy at and around the transcription start site. Spontaneous histone removal or unwrapping is too slow to account for the tight control and regulation of gene expression. ATP-dependent chromatin remodeling complexes facilitate the relocation of DNA sequence elements relative to the histone octamer, thus playing a critical role in gene regulation [42]. Moreover, recent studies have shown that diverse ATP-dependent remodeling complexes confer a repositioning specificity directed at least in part by DNA sequence information [43].

### ATP-Dependent Remodelers Use Primary Sequence Cues To Reposition Nucleosomes

**Figure 7:**
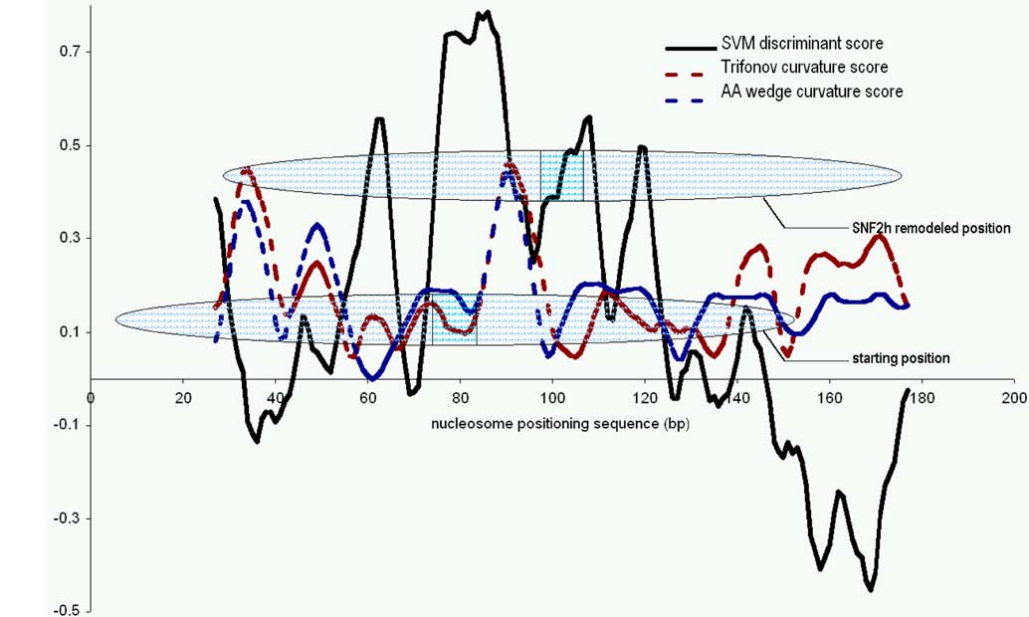
SVM discriminant and DNA curvature scores are consistent with nucleosome position data from in vitro experiments. Solid line indicates A375-trained SVM discriminant score, red and blue dotted lines indicate curvature scores calculated with http://www.lfd.uci.edu/gohlke/curve, using the “Trifonov” and “AAWedges” models, respectively. The lower oval represents the salt dialysis position adopted by the histone octamer on this sequence, while the upper oval represents the SNF2h remodeled position. The dyad axis of the nucleosome is shaded.

We next sought to understand whether the sequence features identified by the SVM are consistent with nucleosome positions adopted in in vitro experiments. The specific locations of nucleosomes along the DNA sequence may play both inhibitory and activating roles in nuclear processes. One way to alter the position of nucleosomes relative to DNA sequence is through the action of ATP-dependent remodelers. These remodelers may use cues from the primary sequence to restrict the remodeling to certain genomic loci or confer a directionality upon the movement of the DNA with respect to the histone octamer. Using the positions of nucleosomes and remodeled products on the 202 bp TPT fragment determined by Fan et al. [44], we sought to understand the characteristics of the starting and remodeled product. We generated SVM discriminant scores for the 202 bp TPT fragment by dividing the sequence into overlapping 50-mers. We then took the resulting discriminant scores and overlaid the starting and remodeled positions of the histone octamer along this sequence. The starting position of nucleosome at the 5′ end of the sequence clearly shows higher discriminant scores, indicating that these are indeed nucleosome-forming sequences (Figure 7, solid line). The dominant starting nucleosome position is centered directly over the 50 bases with the highest scoring discriminant score. In this set of experiments the SVM predicts the starting position of the histone octamer on the DNA sequence.

At 1.5 helical turns on either side of the histone octamer dyad axis, nucleosomal DNA is severely bent to accommodate necessary contacts with the H3/H4 tetramer [38],[45],[46]. Extreme DNA curvature is known to potentiate nucleosome positioning in vitro and under physiological conditions [47],[48]; however, such extreme curvature is likely to be rare in vivo. Computational curvature scores have been explored as predictors of nucleosome positioning [47]–[49]. We therefore compared the SVM discriminant score (Figure 7, solid line) to the DNA curvature scores (Figure 7, dotted lines). The two signals do not correlate, indicating that the SVM scores are picking up on signals that discriminate on features not limited to the bending potential of DNA. Thus, information from both the SVM discriminant scores and DNA curvature predictions may be used to understand primary sequence determinants of nucleosome position. On the TPT+45 the highest scoring curvature region occurs just downstream of the nucleosome dyad axis. Thus, it appears for this sequence that the general placement of the nucleosome can be determined by elements of the primary sequence as recognized by the SVM, and the fine position is determined by the potential to deform the DNA around the histone octamer.

Finally, we investigated how remodeled nucleosome states correlate with SVM score by comparing mapped positions of nucleosome starting positions and their remodeled products on the 202 bp TPT fragment [44]. Recently, Rippe et al. [43] proposed a model in which the remodeling activities of different remodelers depends upon sequence features of the DNA sequence of the nucleosome. The Sf2h remodeled position from Fan et al. [44] occupies regions of high SVM discriminant score and shifts the DNA fragment of high curvature to the region just upstream of histone octamer dyad axis. The nucleosome is translationally repositioned to the closest curvature amenable position within the context of the highest scoring region of nucleosome occupancy as predicted by the SVM discriminant scores. Thus, it appears that the primary DNA sequence signals, as determined by curvature and SVM discriminant scores, may be used to predict and understand nucleosomal occupancy of both starting and remodeled nucleosomal states.

## Discussion

Our results suggest that the human genome contains sequence-based signals that contribute to the placement of nucleosomes. By focusing only on the top- and bottom-ranked probes in microarray datasets, we operate under the hypothesis that only a subset of nucleosomes are well-positioned. This hypothesis agrees with the statistical positioning theory of nucleosome organization and is supported by two previous studies in yeast, each of which concluded that a sequence-based model explains only 15% more nucleosome positioning than is explained by a random model [11],[13]. We have shown, using cross-validation on two independent datasets, that a subset of human nucleosome positions can be accurately predicted using an SVM trained only on sequence patterns.

Additionally, the model of nucleosome occupany affirms earlier work suggesting that DNA sequences shorter than the 150 bp canonically protected by a nucleosome may make a significant contribution to nucleosome occupancy [8],[50]. Great strides have recently been made in understanding primary sequence determinants of nucleosome formation in yeast and worm models [11],[51]. Each of these experiments has used entire core sequences from nucleosome that occupy a constant genomic location: the same protection of approximately 150 bases is occupied by the nucleosome in all cells. However, a relatively small percentage of nucleosomes are well positioned in eukaryotic genomes [51]. Our model is trained using primary sequence features of occupancy alone and does not preselect for nucleosomes that occupy a single position in the genome. This may account for the discrepancies between yeast occupancy models and our human model. Our strategy allows for the analysis of short sequence features that determine the nucleosome-forming or inhibitory potential of a given short sequence of DNA.

The likelihood that a short sequence of DNA will be incorporated into a nucleosome depends upon two features of the DNA: the ability of the sequence to form specific interactions with the histone core and a sequence dependent DNA curvature that allows it to wrap around the histone core. Seminal work by Drew and Travers [29] addressed DNA bending and its relation to nucleosome position. Since then, rules and codes for nucleosome positioning have been defined in terms of the intrinsic flexibility of a particular sequence of DNA. Herein, we show that our model accurately identifies the correct nucleosome position on a nucleosome positioning sequence. Moreover, we show that the model recognizes something other than DNA curvature signals. Thus, our model of nucleosome occupancy may be combined with curvature predictions to better understand and predict locations of nucleosomes and how nucleosomal occupancy may be altered by ATP dependent remodelers.

We observed a complementarity in the SVMs trained using strongly versus weakly digested DNA samples. SVMs trained from weakly digested samples, such as the A375 sample from Ozsolak et al. or the data of Dennis et al., accurately predict regions of strong protection from MNase cleavage, which presumably correspond to well-positioned nucleosomes. These models also correlate strongly with two previously described models of nucleosome positioining in yeast. In contrast, SVMs trained from more completely digested DNA samples, such as the MEC data from Ozsolak et al., are very good at recognizing positions of increased MNase cleavage. These occur, for example, in promoter regions, where strong nucleosome placement would presumably impede the transcriptional apparatus.

Nucleosomal occupancy and chromatin structure have functions in the regulation of transcription [42]. The concept that promoters and classical *cis*-regulatory elements such as enhancers represent nucleosome-free regions was well-established in the literature by the mid-1980s, and derived from chromatin structure assays performed on hundreds of eukaryotic genes from a variety of species [52]. Multiple reports using genomic technologies have recently emerged in support of the generality of this principle across the yeast genome [17],[19],[41],[53]. The low SVM discriminant scores upstream of the TSS indicate that primary sequence features play a role in the regulatory nature of chromatin. These low occupancy scores point toward roles that the primary sequence may play in chromatin remodeling (i.e., the transient repositioning of nucleosomes) and histone eviction. Additionally, recent studies of chromatin remodeling have shed considerable light on the relationship between nucleosome occupancy and competitive interaction with regulatory factors [54]–[57]. Our results suggest a powerful role for the SVM in prediction of nucleoseome-disfavoring sequences, which are fertile ground for regulatory protein interactions.

An advantage of our models of nucleosome occupancy is that we are able to scan for possible nucleosome-free regions in a manner that is unbiased by genomic location or regulatory factor binding. Indeed, multiple laboratories have shown well-positioned nucleosomes flanking regulatory nucleosome-free regions [14],[58]. Thus, the model of nucleosome occupancy may act as a tool for the de novo prediction of regulatory regions within the human genome.

Taken together, our results suggest that we have developed a potentially powerful model of short-range nucleosomal organization that can predict the location of genomic regions that may have an intrinsic predisposition to harboring *cis*-regulatory elements. Recent work [59] suggests that the nucleosome data of Yuan et al. contains significant long-range correlations. Consequently, a future model that explicitly includes these correlations might successfully capture features of nucleosome organization at a larger scale than were examined in the current study.

We emphasize that our results are fundamentally consistent with the long-standing statistical positioning theory of nucleosome organization, which posits that nucleosome positioning is largely secondary to strong non-nucleosomal boundary events. Our model may be used to generate novel testable hypotheses concerning the role of nucleosome-DNA interactions in transcription and other chromosomal regulatory processes.

## Methods

### Identifying Top- and Bottom-Ranked Probes

The Dennis dataset contains three arrays with each probe spotted three times in each orientation, yielding a total of 18 measurements for each of 56,633 50-mer loci. To identify top- and bottom-ranked probes, we follow a five-step procedure. First, we eliminate all probes that overlap a repetitive element as identified by RepeatMasker. Second, we convert the log-ratio intensity values on each array into ranks. Third, for each locus and each strand, we sum the corresponding nine ranks. Fourth, we sort these rank-sum values into a single list. Fifth, we move a threshold down this list from highest to lowest values, accepting into the list of top-ranked probes any probe whose forward and reverse rank-sums are above the current threshold. We stop the traversal when 1,000 top-ranked probes have been identified. The bottom-ranked probes are identified in a similar fashion, traversing the list in the opposite direction.

Top- and bottom-ranked probes in the Ozsolak data are identified using a different rank-based procedure. The Ozsolak dataset contains seven different cell types. Initially we compute a single value for each probe: for IMR90 and MALME with three samples, this single value is the median; for A375 with two samples, the single value is the mean; the remaining four cell types (MEC, MCF7, PM, T47D) have a single sample each. To identify top-ranked probes, we then produce a single sorted list containing all of these values. Traversing this list from largest to smallest value, we accept a probe into our list of top-ranked probes when 5 of the 7 cell types have been observed in the sorted list, as long as no probe within 50 bp has already been accepted into the list. We continue this procedure until 1,000 probes have been accepted. The list of bottom-ranked probes is derived in a similar fashion, traversing the list in reverse order.

### Feature Vectors

Prior to analysis by the SVM, each 50-mer probe is converted into a vector in which the entries are normalized counts of occurrences of all possible *k*-mers for *k* = 1···6. Combining reverse complements leads to a total of 2,772 entries in this vector. For each value of *k*, the corresponding counts are normalized so that the sum of their squares is 1. Thus, the final vector resides on a sphere with radius 6 in a 2,772-dimensional space.

### Support Vector Machines

SVMs are trained using svmvia (http://noble.gs.washington.edu/proj/svmvia), which implements the entire regularization path optimization algorithm [60]. For each training set, the regularization parameter is selected to maximize the ROC score computed on a hold-out set from within the training set. The SVM uses a linear (dot product) kernel.

### Wavelet Smoothing of Microarray Data

Smoothed microarray values are generated using wavelets. The maximal overlap discrete wavelet transform (MODWT) [61] is applied to raw replicate array values from each array separately. Given the input resolution of approximately 20 bp, we construct *j*th-level smoothing for *j* = 2 and 3, giving an output smoothing window of approximately 80 and 160 bp, respectively. The *j*th-level smooth is constructed using the MODWT multi-resolution analysis using the “la-8” wavelet family. Calculations are performed in R using the waveslim library http://cran.rproject.org/src/contrib/Descriptions/waveslim.html.

### Identifying Sites of High and Low Predicted Occupancy

Predicted highly occupied positions are identified by ranking the ENCODE 50-mers by SVM discriminant, and then traversing the ranked list from highest to lowest, accepting any 50-mer that is not marked as repetitive or low complexity and that is more than 250 bp away from any previously accepted prediction and continuing until 1,000 50-mers have been accepted. A similar procedure is carried out in reverse to identify sites of low occupancy.

## Supporting Information

Figure S1 Similar to Figure 5, but the panels were generated using top- and bottom-scoring probes from each of the SVMs.
(0.53 MB PDF)
