# Supplementary material for: Predicting Human Nucleosome Occupancy from Primary Sequence
Source: PLoS Comput Biol. 2008 Aug 22;4(8):e1000134. doi: 10.1371/journal.pcbi.1000134 (PMC2515632; doi:10.1371/journal.pcbi.1000134)
Supplement: Figure S1 — Similar to Figure 5, but the panels were generated using top- and bottom-scoring probes from each of the SVMs. (0.53 MB PDF) [file pcbi.1000134.s001.pdf]

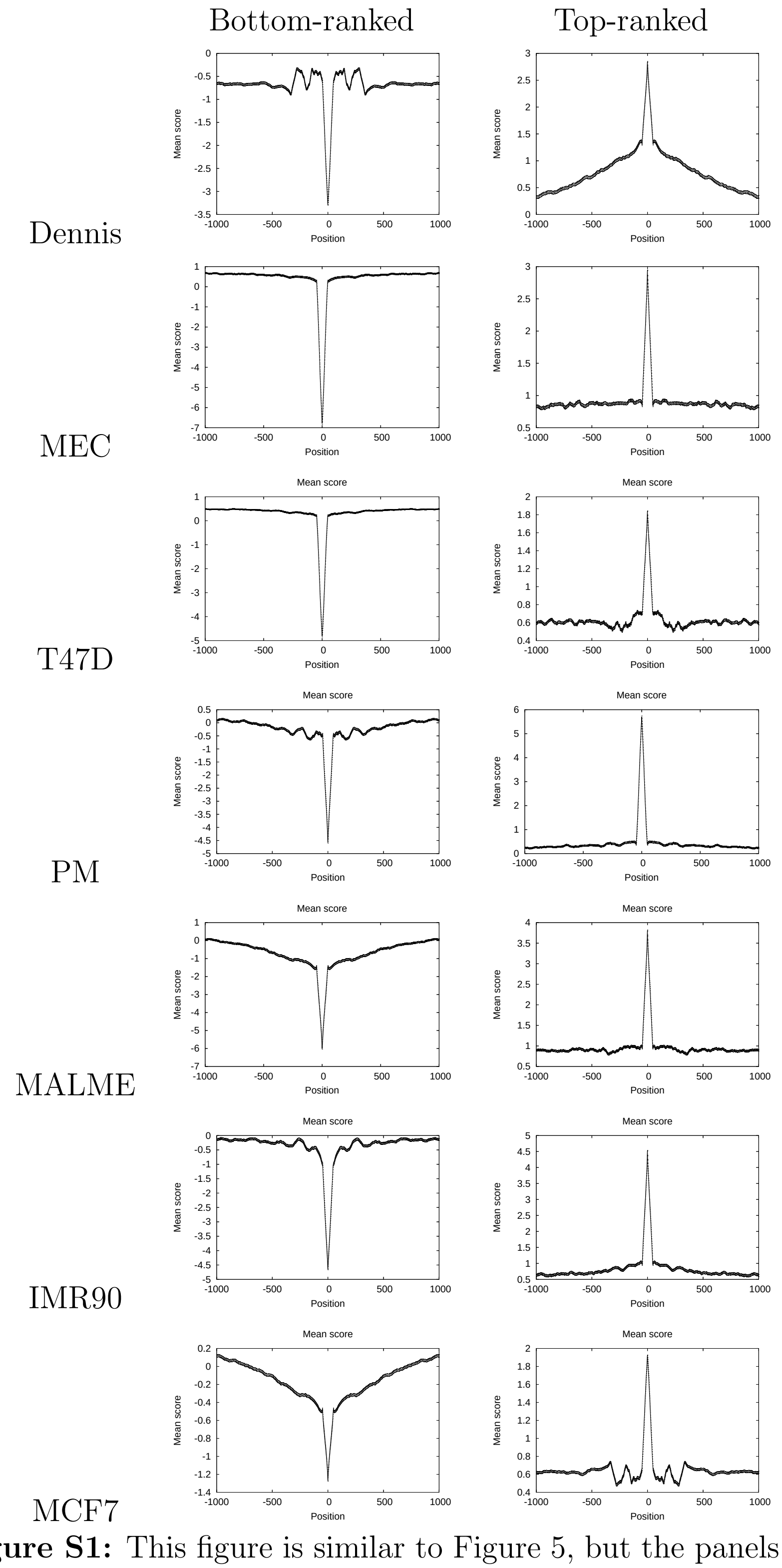

Figure S1: This figure is similar to Figure 5, but the panels were generated using top- and bottom-scoring probes from each of the SVMs.
